# Developing a Polygenic Risk Score for Weight Gain in People Treated for Psychosis—Application in a Real‐World Setting

**DOI:** 10.1002/hup.70035

**Published:** 2026-03-14

**Authors:** Adrian Heald, Yasitha Illangasekera, Camila Marcelino Loureiro, Mark Shakespeare, Adam Jameson, Adrian Phillipson, Gavin P. Reynolds, Caroline Dalton

**Affiliations:** ^1^ Department of Endocrinology and Diabetes Salford Royal Hospital Salford UK; ^2^ The School of Medicine and Manchester Academic Health Sciences Centre Manchester University Manchester UK; ^3^ Sheffield Hallam University Biomolecular Sciences Research Centre Sheffield UK; ^4^ Department of Pharmacology, Faculty of Medicine University of Peradeniya Peradeniya Sri Lanka; ^5^ Rotherham, Doncaster and South Humber NHS Foundation Doncaster UK; ^6^ School of Pharmacy and Medical Sciences University of Bradford Bradford UK; ^7^ Department of Pharmacy Bradford District Care NHS Foundation Trust Bradford UK

**Keywords:** antipsychotic, association, gene, polygenic risk score, psychosis, weight gain

## Abstract

**Introduction:**

Genetic factors are thought to play an important role in antipsychotic‐induced weight gain (AIWG). Polygenic risk scores (PRS) could provide a measure of genetic predisposition to antipsychotic drug induced weight gain (AIWG).We conducted a study to examine how a PRS, generated using SNPs, identified from a recent meta‐analysis, related to weight‐change over time in people with first episode‐psychosis.

**Methods:**

The PRS included SNPs in six different genes, identified as having significant associations (*p* < 0.05) with AIWG. These were *HTR2C* rs3813929; *MTHFR* rs1801133; *ADRA2A* rs1800544; *MC4R* rs489693; *LEPR* rs1137101 and *CNR1* rs1049353. An additive PRS and a risk allele based weighted PRS were created based on risk allele counts and presence or absence of risk alleles respectively. The additive PRS was also used to create low/high genetic risk groups for analysis. The association between PRS and weight gain per day (WGPD) in grams/day as well as BMI percentage change (=> 7%) was investigated using regression models.

**Results:**

In multiple regression analysis, the additive PRS significantly predicted AIWG in females (adjusted *r* ^2^ = 0.59, B: unstandardised regression coefficient = 24.4 g/day *p* < 0.05), but not in males. ANCOVA showed that high genetic risk groups had greater WGPD (*p* = 0.018), with significant PRS gender interactions driven by markedly higher WGPD in high‐risk females (*p* = 0.039). None of the models tested were associated with BMI percentage change.

**Conclusion:**

We report a PRS that is predictive of weight gain in women treated for first episode psychosis, accounting for 59% of the variance daily weight‐gain over time. Validation in an independent cohort is required, as is determining whether it is feasible to apply the PRS prospectively.

## 1 Introduction

Antipsychotic‐induced weight gain (AIWG) is a common and clinically important adverse effect of treatment, with most antipsychotics being associated with an increased risk of weight gain (Barton et al. 2020). This effect is greatest in the first months after initiation of antipsychotic treatment (A. Heald et al. 2025; A. H. Heald et al. 2025).

There is marked variation in the degree of weight gain and metabolic consequences between individuals treated with antipsychotics in both the intermediate and longer term (Cooper et al. 2016). Genome‐wide association studies have identified genes associated with obesity in the general population (Fall and Ingelsson 2014). There is consistent evidence that genetic variants in multiple biological pathways ‐ including neurotransmitter systems, metabolic regulation and energy balance can influence the occurrence of antipsychotic drug side effects including weight gain (Zhang and Malhotra 2011). Furthermore, alterations in appetite regulating hormones and neuropeptides, cell signalling molecules, neurotransmitter‐receptor interactions and gut microbiome have been proposed as mechanism for this (Silas et al. 2025).

A recent meta‐analysis of published evidence identified 6 genetic variants ‐*HTR2C* rs3813929; *MTHFR* rs1801133; *ADRA2A* rs1800544; *MC4R* rs489693; *LEPR* rs1137101 and *CNR1* rs1049353, which predispose individuals to weight gain associated with antipsychotic treatment in adults, five of these having a role in the hypothalamic regulation of appetite and satiety (A. H. Heald et al. 2025b). A previous meta‐analysis (Zhang et al. 2016) reported 13 SNPs from nine genes to be associated with weight gain in children and adults treated with antipsychotics; this showed significant overlap with the more recent meta‐analysis.

Increasing amounts of genetic data have led to the development of polygenic risk scores (PRSs) for a variety of disorders (Slunecka et al. 2021).These scores aggregate the effects of multiple genetic variants into a single measure, aiming to stratify individuals based on their inherited genetic risk of developing various common disorders. Applied in a clinical context, PRSs aim to optimise individual patient treatment and management.

PRS studies have become popular in psychiatric research focussing on various psychiatric disorders, including schizophrenia, with studies investigating the associations of PRS for schizophrenia with antipsychotic treatment responses as outcomes. Work exploring the link between PRS and metabolic dysregulation has been limited, (Yoshida et al. 2023) suggested that PRS analysis may contribute to identifying risk factors for AIWG. Such an approach may help to elucidate the mechanisms at play in AIWG.

We here examined in a real‐world study, how a PRS may relate to weight change in antipsychotic‐treated people with first episode psychosis (FEP).

## 2 Methods

This was a proof‐of‐concept study to determine whether a PRS is associated with weight gain in patients receiving antipsychotic drugs in a real‐world setting.

The participants in the study were selected in a retrospective survey of consecutive records of individuals receiving antipsychotic medication. Only individuals with a baseline body weight measurement taken up to six months before and up to one month after the commencement of antipsychotic medication known to associate with weight gain were selected for analysis. The minimum duration of antipsychotic treatment was six weeks.

Once selected, individuals were asked to provide a saliva sample for genetic analysis. Recruitment was in 3 mental health trusts in England, Rotherham Doncaster and South Humber NHS Foundation Trust (RDASH), Greater Manchester Mental Health (GMMH) and Bradford District Care NHS Foundation Trust (BDCFT). IRAS reference was 19/SC/036. A favourable ethical opinion was given by the South‐Central Berkshire Research Ethics Committee Reference 19/SC/0364.

### 2.1 Polygenic Risk Score (PRS)

The PRS included SNPs in six different genes, identified as having significant associations (*p* < 0.05) with AIWG (A. Heald et al. 2025; A. H. Heald et al. 2025). These were *HTR2C* rs3813929; *MTHFR* rs1801133; *ADRA2A* rs1800544; *MC4R* rs489693; *LEPR* rs1137101 and *CNR1* rs1049353.

Two PRS scales were constructed assuming (1) additive effects of risk alleles and by (2) dichotomising the analysis based on the number of risk alleles and weighting the risk allele groups from effect sizes of the 6 SNPs derived from the meta‐analysis. These parallel approaches allowed us to test which model better captured the genetic contribution to AIWG. The additive scale was constructed assuming a per allele dose–response effect. Here, each SNP was scored as 0, 1, or 2 according to the number of risk alleles carried. Scores across the six loci were summed to generate a total additive PRS, providing a possible range of 0–12.

The additive PRS was further utilised to create ‘high’ and ‘low’ genetic risk categories, based on the presence of ≤ 6 risk alleles (low risk category) and ≥ 7 risk alleles (high risk category). The second PRS scale—the weighted dichotomised allele effect PRS was constructed by recoding each SNP as 0 (no risk allele) or 1 (one or two risk alleles present). The Hedges *g* value calculated for each SNP in the meta‐analysis was directly utilised to weight the risk allele genotype groups for WGPD outcome analysis. The Hedges *g* values (derived from our meta‐analysis study) for each SNP were used to weight the dichotomised allele effect PRS as per presence or absence of risk allele carrying genotypes are given in Table S1: Appendix Table 1.

For the BMI percentage change outcome analysis, the Hedges *g* was converted to log(OR) values which were used to weigh the risk allele genotypes. In each approach the weighted SNP values were summed to create the weighted PRS scales.

### 2.2 Laboratory Analysis

Genomic DNA from saliva samples was extracted from saliva samples using QIAamp DNA midi kits (QIAGEN, Germany). SNP genotyping was performed by real‐time PCR and allelic discrimination using Taqman assays (Applied Biosystems, Foster city, CA). Real‐time PCR was performed in a 96‐well format in a total 10 µL reaction volume using VIC/FAM dye labelled allelic probes with TaqPath ProAmp Genotyping Master Mix. The reaction mixture was subjected to a standard thermal protocol of 95°C for 10 min, 40 cycles of 95°C for 15 s and 60°C for 1 min in a StepOnePlus Thermocycler (Applied Biosystems, Foster City, CA).

### 2.3 Statistical Analysis

Data analysis was conducted using SPSS v25software. The primary outcome measures investigated were weight gained per day (WGPD, g/day) and BMI change of ≥ 7% versus < 7% from baseline since the start of medications.

The association between predictors and WGPD were assessed using multivariate regression and ANCOVA models whilst associations between predictors and BMI changes were investigated with logistic regression models. The age, baseline weight/BMI were entered as covariates and gender was entered as a cofactor in the regression models.

## 3 Results

Analysis was performed on 41 individuals (20 women) for which a full set of data was available and who were taking an antipsychotic agent known to be associated with weight gain. None of the service users approached for this study declined to provide a saliva sample for genetic analysis.

Details of patient characteristics including prescribed medication are given in Table 1. There were no significant differences between men and women with regards to age, baseline body weight and BMI or PRS values.

**TABLE 1:** Description of study sample and medication prescribed.

Characteristic	Overall (*n* = 41)	Male (*n* = 21)	Female (*n* = 20)	*p*
Age (years)	38.4 ± 10.6	38.6 ± 10.7	38.2 ± 10.8	0.89
Baseline weight (kg)	70.2 ± 16.7	72.0 ± 16.8	68.3 ± 16.8	0.48
Weight change per day (grams/d)	101.2 ± 72.5	106.6 ± 72.4	95.5 ± 74.0	0.63
Additive PRS	7.0 ± 1.9	6.9 ± 1.5	7.1 ± 2.3	0.80
Weighted PRS	397.0 ± 266.1	357.5 ± 225.8	440.7 ± 304.8	0.33

The majority of participants were of White ethnicity (75.6%), with smaller proportions identifying as Black/Black British (7.3%), Asian/Asian British (7.3%), or Mixed Race (4.9%), and 4.9% belonging to other ethnicities or not declaring their ethnic status. With regards to smoking status, just under half of the sample were current smokers (48.8%), while 34.1% were non‐smokers and 14.6% were ex‐smokers.

In total, 20 people were prescribed a single antipsychotic agent over the follow‐up period, 14 people received two antipsychotic agents sequentially, and 7 people received three. Olanzapine was the most frequently prescribed agent (*n* = 28), followed by aripiprazole (*n* = 11), quetiapine (*n* = 7), risperidone (*n* = 6), haloperidol (*n* = 2), clozapine (*n* = 1), and amisulpride (*n* = 1).

### 3.1 Additive PRS Versus WGPD

Multiple regression analyses were conducted to examine the association between the additive PRS and WGPD, adjusting for age and baseline weight (Table 2). In the overall sample, the additive PRS was a significant predictor of WGPD (B: unstandardised regression coefficient = 12.53, *p* = 0.035). Hence, each one‐unit increase in PRS corresponded to 12.5 g increase in weight per day, adjusting for age and baseline weight. The gender stratified analyses indicated that the effect was driven by females, in whom PRS significantly predicted WGPD (B: unstandardised regression coefficient = 24.36, *p* < 0.0001) with the model explaining 59% of the variance in WGPD. A one‐unit increase in PRS corresponded to a 24.4 g/day increase in weight.

**TABLE 2:** Multiple regression predicting weight gain per day from additive polygenic risk score.

Sample	*B*	SE B	*p*	*R* ^2^	Adj. *R* ^2^	F(df1, df2)	Model *p*
Overall	12.53	5.71	0.035	0.19	0.12	2.8 (3, 36)	0.052
Females	24.36	5.22	< 0.001	0.62	0.59	8.3 (3, 15)	0.002
Males	−8.76	11.0	0.437	0.13	−0.02	0.88 (3, 17)	0.469

*Note:* Age and baseline covariates not shown.

Abbreviations: Adj. *R* ^2^, adjusted *R* ^2^; B, unstandardised regression coefficient; df, degrees of freedom; F, F‐statistic; *p*, probability value; *R* ^2^, coefficient of determination; SE B, standard error of B.

In males, the additive PRS was not significantly associated with WGPD. Furthermore, age and baseline weight were not significant predictors of WGPD in any of the models of analysis.

In order to investigate which SNPs of the PRS most strongly predicted WGPD, each SNP was entered separately into a covariate adjusted linear regression model with WGPD as outcome. Based on estimated marginal means following adjustment for multiple comparisons, the MC4R A allele (in females), MTHFR C allele and LEPR G allele were found to be the most significant drivers of the predictive power of the additive PRS.

**FIGURE 1:**
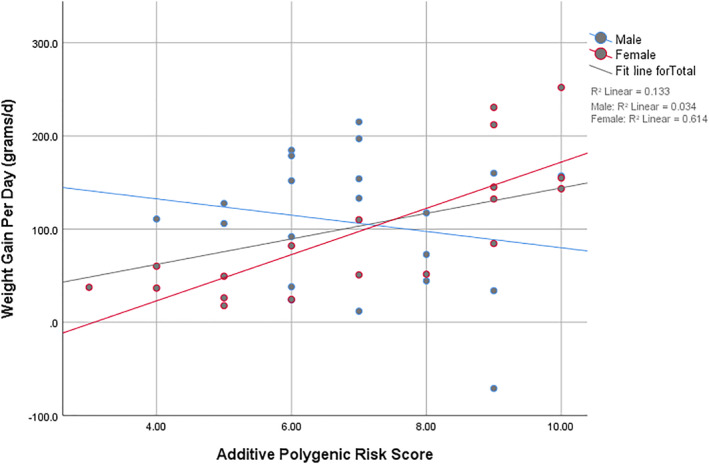
Simple scatter plot of additive polygenic risk score (PRS) versus weight gain per day.

The relative strength of correlations between the additive PRS and WGPD in the overall study group, females and males are shown in Figure 1.

There was no significant association between the additive PRS and BMI percentage change (*p* = 0.87) for men and women confined.

### 3.2 Additive PRS Categories Versus WGPD

An ANCOVA was conducted to examine the association between low and high genetic risk score categories (=< 6 risk alleles vs. => 7 risk alleles) and WGPD, controlling for age and baseline weight, whilst including gender as a factor. The results are shown in Table 3.

**TABLE 3:** Association of high versus low genetic risk categories with weight gain per day.

Source	*F*	*p*
Age	0.12	0.733
Baseline weight	2.92	0.097
PRS category	4.89	**0.039**
Gender	0.94	0.339
PRS category x gender	6.22	**0.018**

Abbreviations: df, degrees of freedom, (1,34); F, F‐statistic.

The overall model was significant, (*p* = 0.021). There was a significant main effect of PRS category such that participants with high risk (≥ 7 risk alleles) had higher adjusted WGPD scores (adjusted Mean = 122.2 g/day, 95% CI [44.9, 109.1]) than those with ≤ 6 risk alleles (adjusted Mean = 77.0 g/day, 95% CI [94.8, 149.6]).

**FIGURE 2:**
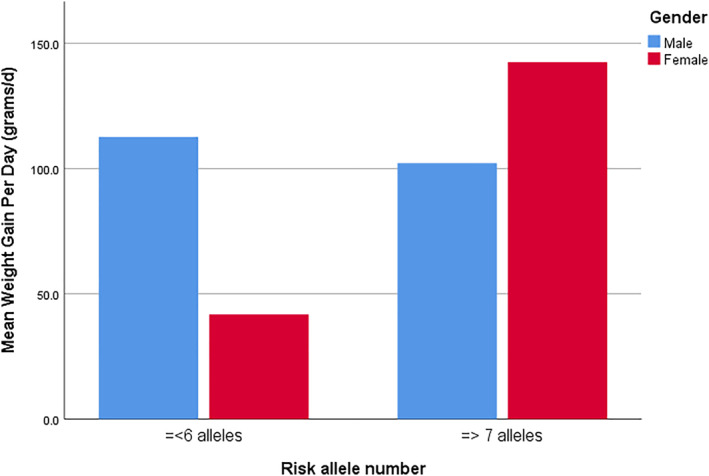
Interaction effect of non‐weighted polygenic risk score and gender on weigh gain.

The main effect of gender was not significant (*p* = 0.339) However, there was a significant interaction between PRS category and gender, F (degrees of freedom; 1,34) = 6.22, *p* = 0.018 (Figure 2). Follow‐up comparisons indicated that in females, those with ≥ 7 risk alleles had substantially higher WGPD scores (adjusted Mean = 138.8 g/day, 95% CI [99.6, 178.1]) compared with those with ≤ 6 alleles (adjusted Mean = 40.4 g/day, 95% CI [−5.5, 86.2]). In males, WGPD scores were not significantly different across genetic risk categories.

### 3.3 Weighted Dichotomised Alleles Effect Derived PRS Versus WGPD

A multiple regression was conducted to examine whether the weighted dichotomised allele effect PRS, age, gender, and baseline weight and total predicted weight gain per day. The overall model was not significant, *F* (degrees of freedom; 4, 36) = 1.21, *p* = 0.321, and none of the predictors were significantly associated with WGPD. Furthermore, the ln(OR) weighted PRS did not significantly predict *a* > 7% BMI change in the study population (*p* = 0.44).

## 4 Discussion

Currently, no genetic risk scores are available for people starting antipsychotic treatment to predict their propensity for weight gain. A clinically relevant and easily applicable tool to enable risk stratification regarding weight gain and early intervention may be helpful. This study aimed to apply the PRS to real world collected data. We found that a higher PRS was associated with weight gain in women treated for first episode psychosis, accounting for 59% of variance in weight gain over time. Gender differences in response to antipsychotic treatments are recognised, with females reported to having a greater risk of AIWG compared to males (A. Heald et al. 2025a; Moniem and Kafetzopoulos 2025).

Preventative treatment has been shown to be more effective than retrospective treatment in mitigating weight gain, which itself can lead to non‐concordance with medication (Waite et al. 2022; Klein et al. 2020). Genetic risk scores may have the potential to predict weight gain associated with antipsychotic treatment in psychosis, particularly in individuals with first‐episode psychosis (FEP) (Muntané et al. 2023). Previous studies have proposed that the weight gain in psychosis is associated with the altered expression of genes related to both obesity and BMI (Crespo‐Facorro et al. 2019), which suggests that there is a genetic overlap between these two medical conditions.

In our study, we modelled the PRS using two approaches: as additive, and by dichotomisation based on the presence or absence of risk alleles. The additive PRS, both when used as a linear scale and when dichotomised to ‘high’ versus ‘low’ risk categories, yielded statistically significant associations with weight gain in females. The additive approach is commonly used in genetic epidemiology because most common variant associations identified through GWAS are best captured by additive effects, reflecting a dose–response relationship in which risk increases with each additional allele.

The weighted allele effect based dichotomised PRS, did not show association with WGPD or BMI percentage change outcomes in our study. By weighting each SNP with the effects sizes derived from the our meta‐analysis, we attempted to construct the PRS which would be applicable to real‐world setting. However, collapsing the heterozygotes and homozygotes into a single category and thusly recoding genotypes into 0 versus 1, substantially reduced the variability of the PRS, particularly when the number of SNPs was modest. This loss of information, potentially attenuated effects and decreased statistical power of the analysis. Considering all above, we believe that the additive model provides a more sensitive and biologically plausible representation of polygenic risk in our study.

It seems that females may carry a greater risk of AIWG due to sex differences in metabolism, body composition, and hormone–neurotransmitter interactions (Silas et al. 2025), though underlying mechanisms remain incompletely understood.

The findings of our study have important clinical implications for the early identification of individuals most vulnerable to weight gain and highlights the importance of examining genetic pleiotropy in the context of medically important comorbidities for predicting future outcomes. This is exemplified by earlier work (Reynolds et al. 2006) which reported that the combination of a *HTR2C* gene promoter region polymorphism with a common and functional polymorphism of the leptin gene, along with initial BMI, accounted for almost 30% of the variance in initial drug‐induced weight gain in people treated for psychosis. A key question is whether it is feasible to apply such a PRS prospectively in the real world setting of an FEP service—early findings do though suggest that patients are keen to see pharmacogenetic information integrated into antipsychotic decision making (Jameson et al. 2024). Other challenges, such as clinician education and training, may also impact the feasibility of implementing an AIWG PRS into clinical practise.

There is rapid weight gain in the first few weeks after commencing antipsychotics (Rummel‐Kluge et al. 2010). The rate of weight gain then gradually decreases, tending to plateau after several months. Factors associated with rapid weight gain in the initial period are younger age, lower BMI, more robust symptom response and increase in appetite (A. Heald et al. 2025; A. H. Heald et al. 2025; Vandenberghe et al. 2015). Rapid weight gain of more than 5% in the first month is a strong predictor of significant long‐term weight gain (Dayabandara et al. 2017) In our sample, there was a single participant who lost about 6 kg of weight over 3 months whilst on antipsychotic treatment with risperidone. This 44‐year‐old male had a high BMI of 29.8 kgm^2^ when he was commenced on treatment and so his weight loss may have been unrelated to his medication. However, excluding this particular subject from the analysis did not have significant effect on the overall findings from the analysis.

Validation of our study findings with prospective replication would be required prior to clinical implementation. Replication could be done in another NHS cohort of patients undergoing initiation of treatment for FEP. A saliva sample would be obtained at or near initiation of therapy for genotyping. A PRS would be constructed using the same six SNPs and the same primary outcome of WGPD used. Prospective weight change measures would be obtained at early, fixed time points better to capture weight gain. Identical modelling strategies including sex stratified analysis should be conducted. Replication in ethnically diverse samples would support conclusions about generalisability.

If validated in independent cohorts, this polygenic risk score (PRS) could be used to for early risk stratification for antipsychotic‐induced weight gain at the point of care initiation. A single saliva or blood sample at point of care would suffice for genotyping the 6 SNPs required for the PRS.

In an everyday clinical setting, the PRS could be applied alongside established clinical risk factors such as baseline weight, body mass index, age, and early weight trajectory. Individuals identified as being at higher genetic risk—particularly females, in whom the strongest association was observed—could be prioritised for appropriate antipsychotic choice and dosing, metabolic monitoring and early preventive measures such as dietary and exercise counselling.

Importantly, the PRS is not intended to determine treatment eligibility, but rather to inform shared decision‐making and guide preventive strategies during the early phase of antipsychotic treatment. Integration of genetic risk information into routine care has the potential to improve early identification of individuals at greater risk of weight gain, thereby reducing long‐term cardiometabolic morbidity associated with antipsychotic use.

### 4.1 Limitations and Strengths

We accept that we only had a small sample of people. The lack of any significant association of weight gain with the PRS in men is surprising and requires further evaluation. Potentially, greater variation in several non‐genetic drivers of weight gain, including medication and dose, diet and exercise, may have reduced the proportion of variance in male weight gain attributable to the PRS. Nevertheless, we have also previously observed and reported on females having a greater risk of antipsychotic induced weight gain compared to males (A. Heald et al. 2025).

Antipsychotic adherence was also not assessed during data collection, a factor that undoubtedly would have impacted the extent to which weight gain occurred across the sample. We also did not specifically assess for the effects of possible gene‐drug interactions, which could vary for each type of antipsychotic agent prescribed.

We accept that this is a preliminary step in the understanding of the way that genetic predisposition may relate to weight gain in people treated for psychosis and that the PRS needs to be tested prospectively in the real world setting of an FEP service in a feasibility study to examine the feasibility of screening prospectively for risk of weight gain.

## 5 Conclusion

We have identified that the PRS was associated with weight gain in people treated for first episode psychosis in a real‐world clinical setting. The finding was unique to females in whom the PRS accounted for 59% of weight gain in multivariate modelling. Validation of the PRS as a predictive factor for weight gain in treatment of FEP in a prospective study is necessary, as is determining whether it is feasible and valuable to apply a PRS to inform clinical pharmacotherapeutic decisions in the treatment of FEP.

## Author Contributions

A.H. conceived the study with Y.I., A.P., G.P.R. and C.D. with contributions in relation to design from A.J., C.M.L. and C.D. G.P.R. and Y.I. led on data analysis. Data extraction was conducted by M.S. and A.J. Y.I. constructed all the tables and figures. All authors contributed to the final version of the manuscript.

## Funding

The authors have nothing to report.

## Ethics Statement

A favourable ethical opinion was given by the South‐Central Berkshire Research Ethics Committee Reference 19/SC/0364.

## Conflicts of Interest

The authors declare no conflicts of interest.

## Supporting information


**Table S1:** Hedges g values (derived from our meta‐analysis study) for each SNP as used to weight the dichotomised allele effect PRS as per presence or absence of risk allele carrying genotypes.

## Data Availability

The data that support the findings of this study are available from the corresponding author upon reasonable request.
