# Supplementary material for: Developing a Polygenic Risk Score for Weight Gain in People Treated for Psychosis—Application in a Real‐World Setting
Source: Hum Psychopharmacol. 2026 Mar 14;41(2):e70035. doi: 10.1002/hup.70035 (PMC12988834; doi:10.1002/hup.70035)
Supplement: Supplementary file 1 — Table S1: Hedges g values (derived from our meta‐analysis study) for each SNP as used to weight the dichotomised allele effect PRS as per presence or absence of risk allele carrying genotypes. [file HUP-41-e70035-s001.doc]

**Appendix**

Table 1:

Hedges g values (derived from our meta-analysis study) for each SNP as used to weight the dichotomised allele effect PRS as per presence or absence of risk allele carrying genotypes.

| **SNP** | **Weights assigned to genotypes (Hedges g from meta-analysis) (Heald et al., 2025)** |
| --- | --- |
| ADR2A rs1800544 | CC+ CG = 0.71, GG=0 |
| HTR2C rs3813929 | TT + CT = 0.69, CC=0 |
| LEPR rs1137101 | GG+GA = 0.18, AA=0 |
| MC4R rs489693 | CC+AC = 0.13, AA=0 |
| MTHFR rs1801133 | CC+CT = 0.31, TT=0 |
| CNR1 rs1049353 | CC+CT = 0.55, TT=0 |
